# Limb-Salvage Reconstruction of the Proximal Humerus Using Patient-Specific 3D-Printed PEEK Implants: A Midterm Clinical Study

**DOI:** 10.3390/bioengineering13020253

**Published:** 2026-02-22

**Authors:** Tran Duc Thanh, Le Duc Huy, Nguyen Duc Trung, Luong Nhat Anh, Vu Duc Thang, Luu Huu Phuc, Le The Hung, Vo Sy Quyen Nang, Pham Trung Hieu, Nguyen Tran Quang Sang, Dang Minh Quang, Tran Trung Dung

**Affiliations:** 1 Sarcoma Center, Vinmec Times City International Hospital, Vinmec Healthcare System, Hanoi 113-900, Vietnam24trung.nd@vinuni.edu.vn (N.D.T.);; 2 Graduate School of Medicine, Osaka Metropolitan University, Osaka 545-8585, Japan; 3 College of Health Sciences, VinUniversity, Hanoi 131-001, Vietnam; 4 3D Technology in Medicine Center, College of Health Sciences, VinUniversity, Hanoi 131-001, Vietnam

**Keywords:** osteosarcoma, proximal humerus reconstruction, PEEK, 3D printing, bone tumor, patient-specific implant

## Abstract

**Background:** Reconstruction of the proximal humerus after wide tumor resection is technically demanding, and traditional methods such as allograft–prosthetic composites, reverse shoulder arthroplasty, and metal implants are limited by graft unavailability, pediatric size mismatch, their high cost, and metal-related stress shielding. Polyether ether ketone (PEEK), with its modulus closer to cortical bone and radiolucency, offers a promising alternative. Building upon the success in craniomaxillofacial surgery and its favorable physical characteristics, we applied personalized 3D-printed PEEK implants for proximal humerus reconstruction. This study reports the first evidence of applying patient-specific 3D-printed PEEK implants in the proximal humerus. **Methods:** A retrospective cohort study was conducted on seven patients who underwent wide resection of primary malignant bone tumors of the proximal humerus, followed by reconstruction using patient-specific 3D-printed PEEK implants. Implant design was based on preoperative computed tomography (CT) imaging, incorporating contralateral humeral mirroring and computer-aided design. The implants were fabricated using fused deposition modeling (FDM) with medical-grade PEEK under stringent thermal control (nozzle temperature > 400 °C and heated build chamber), followed by a controlled annealing process to minimize internal stress, optimize polymer crystallinity, and enhance mechanical durability. Outcomes assessed included implant survival, oncologic control, shoulder range of motion, and functional outcomes measured using the Musculoskeletal Tumor Society (MSTS) score. The mean follow-up duration was 56.3 months. **Results:** All patient-specific PEEK implants were successfully manufactured and implanted with satisfactory geometric accuracy. Mechanical implant survival was 85.7% at final follow-up, with one implant fracture occurring at 28 months. No cases of deep infection, dislocation, loosening, or permanent neurovascular injury were observed. Local soft-tissue recurrence occurred in two patients (28.6%), without distant metastasis or tumor-related mortality. The limb-salvage rate was 100%. At final follow-up, the mean MSTS score was 23.0 ± 1.6. Shoulder motion was limited but comparable to outcomes reported for conventional anatomic megaprosthetic reconstructions. **Conclusions:** Patient-specific 3D-printed PEEK implants provide a feasible and oncologically safe option for proximal humerus reconstruction after tumor resection, with acceptable midterm implant survival and functional outcomes. The favorable elastic modulus and radiolucency of PEEK offer distinct biomechanical and imaging advantages over metallic implants. Further design optimization and larger prospective studies are warranted to enhance mechanical durability and functional restoration.

## 1. Introduction

The proximal humerus is a common site for primary and metastatic bone tumors, accounting for 7–10% of bone sarcomas. It is the second most frequent site for osseous sarcomas and the third for osteosarcomas. After the femur, the humerus is the second most affected long bone for pathological fractures, with an incidence of 16–27%, significantly impacting load-bearing and daily activities [1,2,3]. Shoulder reconstruction aims to restore stability, preserve deltoid and rotator cuff function when possible, and achieve pain-free mobility to maximize quality of life after major bone loss [4].

Surgical treatment for bone tumors largely depends on tumor histology and typically involves wide resection, often resulting in significant bone defects. However, recent advancements in adjuvant therapies, including multi-target chemotherapy and localized radiation, have enabled an increasing number of patients to undergo limb-sparing tumor resections [5,6,7,8].

There is no consensus regarding the best reconstructive technique after proximal humerus resection. Several reconstructive options are available, including allografts, alloprosthetic composites (APC), megaprosthetic (megaendoprostetic) replacement (MPR), and reverse shoulder arthroplasty (RSA) [9].

Moreover, several autologous grafts (fibula, scapular crest, or clavicle) have been described. Because autologous grafts often are used in conjunction with a shoulder arthrodesis, prostheses, osteo- articular allografts, and allograft-prosthesis composites are the only reconstructions allowing for a mobile glenohumeral joint [10,11,12]. Although all of these approaches are in use, and there are some situations where only one approach might be appropriate for a particular patient, there are many scenarios in which all are potential options. However, because there are no prospective or randomized trials, it is difficult to know which approach is best in terms of functional out- come, implant survivorship, or complications.

Metallic tumor prostheses, while mechanically robust, are characterized by a high elastic modulus which contributes to stress shielding, periprosthetic bone loss, and implant-related complications, particularly in pediatric and young adult populations. In addition, metallic implants generate substantial imaging artifacts, complicating postoperative surveillance for local recurrence, in skeletally immature patients, these problems are compounded by small bone size.

Polyether ether ketone (PEEK) has emerged as a high-performance biomaterial with properties that may address several of these challenges. Advances in three-dimensional (3D) printing now make it possible to combine PEEK’s material advantages with exact anatomic matching. Additive manufacturing can fabricate a patient-specific implant directly from the patient’s Computerized Tomography (CT) or Magnetic Resonance Imaging (MRI) model, yielding a prosthesis that conforms precisely to the resected proximal humerus [13].

PEEK is a member of the Polymers class of biomaterials. Polymers consist of many repeating units of a primary sequence, i.e., monomer, which consists of a long backbone carbon chain linked by covalent bonds. PEEK is classified as a linear homopolymer, i.e., similar monomer segments. PEEK is among the newer special thermoplastic engineering plastic types and has shown promising biological, mechanical, and chemical properties [14,15].

In comparison to metals, PEEK and polymers generally have an elastic modulus closer to that of bone, thereby decreasing the stress shielding effect seen in metals used for arthroplasty or fracture fixation such as stainless steel. They also avoid the side effect of releasing metal ions in the body which may cause adverse effects such as osteolysis and immune reactions. They are more compatible with most radiological techniques such as CT and MRI scans used for the monitoring and follow-up of treatment [16].

PEEK specically has been shown to have superior properties such as high thermal stability, toughness, and rigidity, creep resistance, ease of processing, self-lubricating, and abrasion resistance [16]. It has also been shown to be relatively non-toxic with wear particles that do not damage human cells [17].

To date, however, no PEEK implants in clinical practice for shoulder reconstructions have been reported.

Accordingly, we developed patient-specific 3D-printed PEEK implants by scientist, engineer, and orthopedic doctors. The aim of this study was to evaluate midterm implant survival, shoulder function, and oncologic safety in this novel cohort. We assessed functional performance (MSTS scores, range of motion) and complications (implant failure, tumor recurrence, etc.) to determine whether patient-specific PEEK implants can provide durable, high-function reconstruction without the stress-shielding or imaging limitations of conventional metal prostheses.

## 2. Materials and Methods

This study was conducted using retrospective data. All patients were informed of the potential risks associated with 3D-printed humerus replacement surgery, including the possibility of requiring a second-stage procedure. This study received approval from the Ethics Committee. Written informed consent for the publication of anonymized patient information was obtained for all cases in this study.

### 2.1. Inclusion and Exclusion Criteria

Patients were included in this study if they met all the following criteria: histologically confirmed primary bone sarcoma involving the proximal humerus, indication for wide oncologic resection of the proximal humerus, reconstruction using a personalized 3D-printed PEEK implant, minimum postoperative follow-up of 12 months.

Exclusion Criteria: metastatic bone disease or non-sarcomatous tumors of the proximal humerus, insufficient bone quality or severe osteoporosis.

### 2.2. 3D-Printed PEEK Implant Production

**Figure 1:**
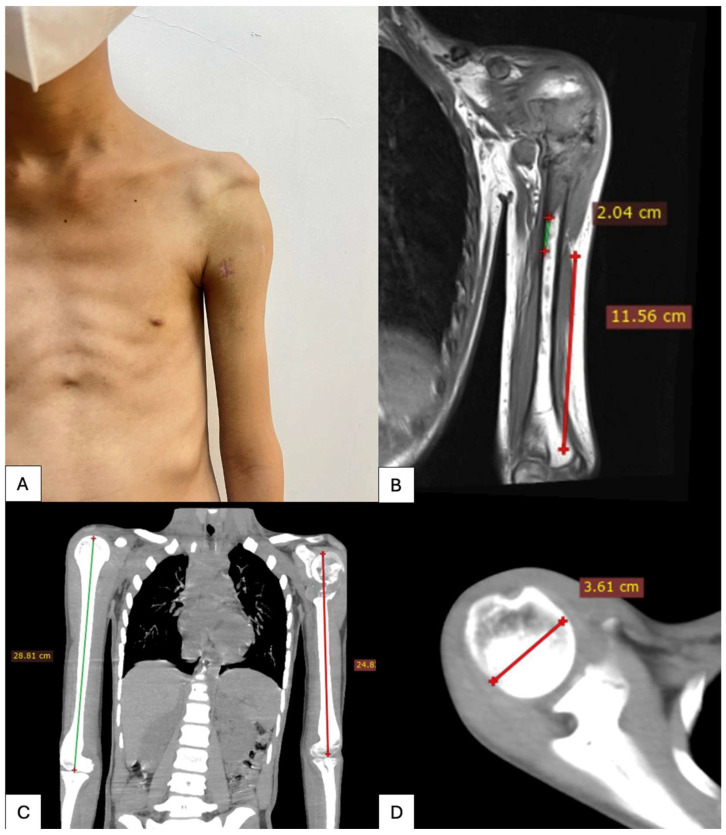
Preoperative imaging and planning for reconstruction and virtual tumor resection. (**A**) Clinical photograph of the patient’s left shoulder. (**B**–**D**) Measurements of humeral length and width on both sides.

All patients underwent preoperative imaging with thin slice computed tomography of the affected shoulder. Accurate CT data acquisition was considered essential for the design of patient-specific implants. CT scans were obtained with a slice thickness of 1.0 mm (Siemens SOMATOM series, Germany), which provides an optimal balance between spatial resolution and image noise while limiting radiation exposure (Figure 1).

Digital Imaging and Communications in Medicine (DICOM) data were imported into Mimics^®^ software (version 28.0, Materialise, Leuven, Belgium) for three-dimensional reconstruction of the proximal humerus. Segmentation was performed to delineate cortical and cancellous bone, and a three-dimensional bone model of the affected humerus was generated. In cases of extensive tumor-related bone destruction, CT data from the contralateral healthy humerus were acquired, mirrored, and used as an anatomical template. Surface-based registration using an Iterative Closest Point algorithm was applied to align the mirrored model with the affected side, allowing restoration of native humeral length, offset, and proximal geometry.

**Figure 2:**
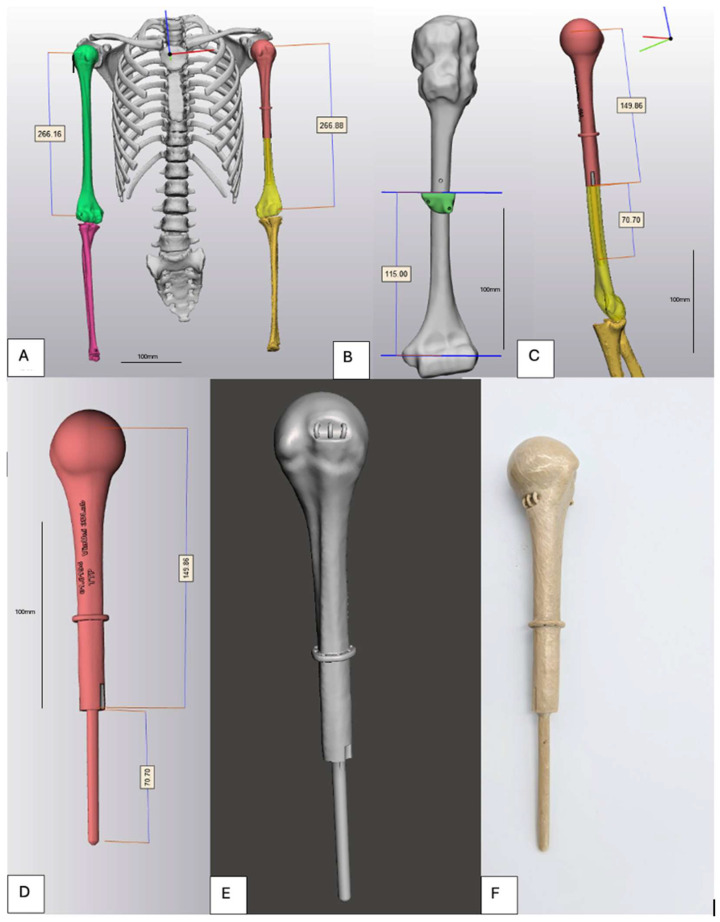
Computer-aided design and fabrication of the 3D-printed PEEK implant. (**A**) 3D reconstruction of both humeri shows similar lengths for contralateral mirroring. (**B**) Residual humeral bone stock after resection for stem length and fixation planning. (**C**) Virtual assembly confirms overlap, alignment, and restored length. (**D**) Implant dimensions: segment and intramedullary stem. (**E**) Implant features include a junctional collar and proximal suture tunnels for stability and soft-tissue reattachment. (**F**) Fabricated implant showing the head–shaft–stem construct.

Virtual tumor resection was performed in the computer-aided design (CAD) environment using mediCAD software (version 7.0, mediCAD Hectec GmbH, Altdorf, Germany) according to preoperative oncologic planning and intended surgical margins. Based on the residual bone morphology, a patient-specific implant was designed using CAD software. The implant consisted of an anatomically contoured proximal segment and a diaphyseal intramedullary stem. Stem diameter and length were individually determined based on the intramedullary canal dimensions of the residual humeral shaft to achieve press-fit fixation while preserving cortical bone integrity. Multiple suture tunnels (2.5–3.5 mm in diameter) were incorporated into the proximal portion of the implant to allow reattachment of the rotator cuff remnants, deltoid muscle, and joint capsule. The implant surface was designed with a smooth finish to reduce soft-tissue irritation (Figure 2).

The finalized implant design was manufactured using medical-grade PEEK by fused deposition modeling (FDM) on a PEEK-300 printer (CreateBot, Zhejiang, China). Printing was performed on a high-temperature industrial FDM printer capable of processing PEEK, equipped with a nozzle temperature exceeding 400 °C and a heated build chamber (≥120–150 °C) to ensure adequate interlayer adhesion and dimensional stability. A controlled printing environment was maintained throughout the fabrication process to minimize warping and internal stress.

Following printing, the implants underwent a controlled annealing process, consisting of gradual heating and slow cooling under predefined temperature conditions, to optimize polymer crystallinity, reduce residual stress, and enhance mechanical strength and fatigue resistance. Post-processing included removal of support structures, surface finishing, and dimensional inspection to verify geometric accuracy relative to the digital CAD model.

The total manufacturing time from finalized DICOM data to completion of the implant was approximately 7–14 days, followed by 48 h of standard medical sterilization prior to implantation. All implants were inspected for dimensional accuracy and surface integrity before surgical use. After they were made, the implants were placed in an autoclave at 121 °C for 30 min, then gamma-irradiated.

### 2.3. Surgical Technique

All the surgeries were performed by a team including experienced musculoskeletal oncologists (T. T. D., N. T. Q. S., T. D. T. and D. M. Q.). After general anesthesia, all patients were placed in the beach chair position. All operations were performed via a deltopectoral approach. The excision of soft tissue depended on the involvement of the tumors. The axillary nerve was identified and protected carefully during the operation. A jigsaw was used to perform the osteotomy according to the preoperative plan. The previous biopsy track along with the tumor was removed.

**Figure 3:**
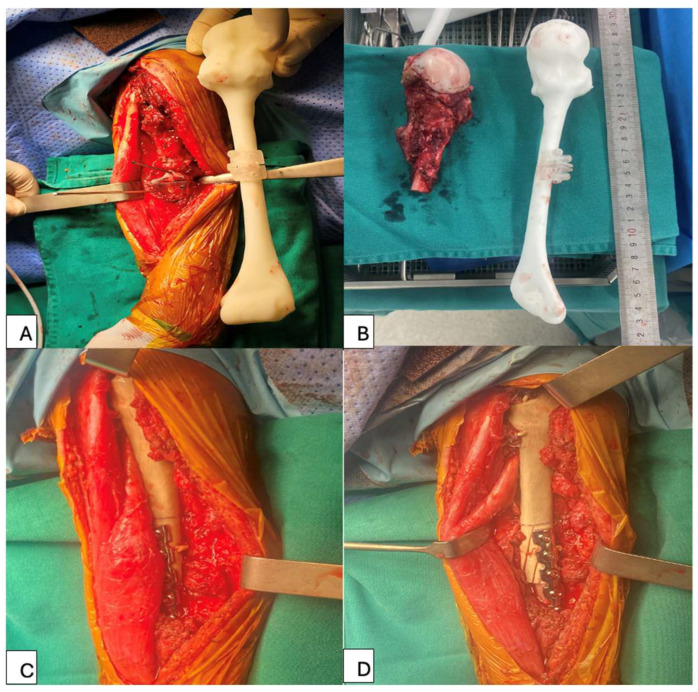
Intraoperative photographs demonstrating wide tumor resection and implantation of the 3D-printed PEEK prosthesis. (**A**) shows the use of a patient-specific instrument (PSI) to determine the tumor resection level during surgery; (**B**) reveals the resected specimen after removal; In (**C**,**D**), the PEEK implant is shown after implantation in the patient’s humerus, viewed from two different positions.

The patient-specific 3D-printed PEEK implant was subsequently implanted into the humeral shaft. Based on preoperative planning, fixation was achieved using a press-fit stem, with or without supplemental polymethylmethacrylate bone cement (Simplex^®^ P, Stryker, MI, USA), and further reinforced with plate-and-screw fixation. The implant was oriented to restore appropriate humeral version and shoulder alignment (Figure 3).

Reconstruction of the soft tissues was performed using the integrated suture tunnels designed into the proximal portion of the implant. Attention was paid to reattachment of the deltoid to optimize postoperative shoulder function. After confirming implant stability and soft-tissue balance, the wound was thoroughly irrigated with saline solution.

### 2.4. Postoperative Management

The shoulder joint was maintained on an abduction splint for at least 6 weeks postoperatively. All patients were allowed to perform active movements of the wrist and the elbow and passive exercises of the shoulder on the first day after surgery. After 6 weeks, patients were encouraged to perform active exercises of the shoulder joint to achieve more flexible ROM.

**Figure 4:**
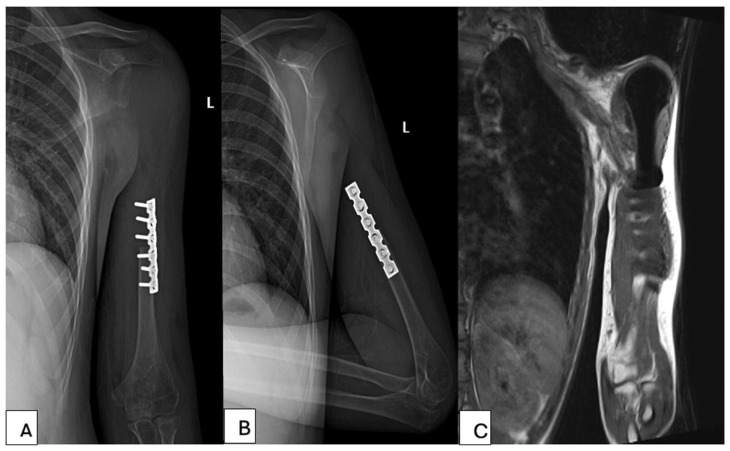
Radiographic follow-up of the patient at 6 months postoperatively. (**A**,**B**) show the patient’s anteroposterior and lateral radiographs; (**C**) shows the postoperative MRI obtained during follow-up evaluation.

All patients were followed up with clinical and imaging assessments. X-ray of the reconstructed shoulder joint was performed after surgery and then every 3 months thereafter. Chest CT scan was performed every 3 months, and bone scan every 6 months to identify potential metastases. At the last follow-up, active ROM, including abduction, forward flexion, external rotation and internal rotation, was measured and recorded. The MSTS functional score were also assessed at the last follow-up (Figure 4).

## 3. Results

### 3.1. Patient and Tumor Characteristics

Seven patients underwent wide resection of the proximal humerus followed by reconstruction using patient-specific 3D-printed PEEK implants. The mean age at surgery was 23 years (range: 7–58 years); five patients were male and two female. The underlying diagnoses included osteosarcoma (*n* = 4, 57.1%), chondrosarcoma (*n* = 2, 28.6%), and Ewing sarcoma (*n* = 1, 14.3%). All tumors were classified as Enneking stage IIB. The tumor involved the right humerus in three cases and the left in four.

Two patients (28.6%) had undergone inappropriate biopsy or unplanned surgery before referral, and two (28.6%) presented with pathologic fractures. The mean follow-up duration was 56.3 months (range: 19–112 months) (Table 1).

### 3.2. Design, Manufacturing, and Geometric Fidelity

All seven patient-specific implants were successfully designed using contralateral mirroring and segmental defect reconstruction, then manufactured via high-temperature FDM of medical-grade PEEK followed by annealing. Post-manufacturing quality control confirmed that all implants met the predefined geometric tolerance, with a maximum surface deviation of less than 2 mm compared with the original CAD model. No gross dimensional mismatch was recorded between planned and produced constructs (Table 2).

### 3.3. Surgical Complications

Three patients (42.8%) experienced complications related to the procedure or implant. Two patients (28.6%) developed local soft-tissue recurrence within the operative field. One patient (14.3%) sustained an implant fracture 28 months postoperatively after a traumatic fall. No cases of dislocation, deep infection, clinically significant loosening, vascular injury, or permanent major nerve injury were observed.

Mechanical implant survival, defined as absence of fracture or loosening, was 85.7% (6/7) at final follow-up. All patients, including the one who underwent revision for implant fracture, maintained limb salvage, resulting in a 100% limb-salvage rate.

### 3.4. Oncologic Outcomes

Two patients (28.6%) developed soft-tissue local recurrence, both managed without amputation. No patient developed distant metastasis during the observation period. At latest follow-up, six of seven patients (85.7%) had no evidence of disease (disease-free), while oncologic status was indeterminate in one patient due to incomplete follow-up data. There were no tumor-related deaths in this cohort (Table 3).

### 3.5. Functional Outcomes

At final follow-up, the mean MSTS score was 23.0 ± 1.6 (range, 21–25). Analysis of shoulder range of motion demonstrated a mean flexion of 35.7°, extension 20.0°, abduction 30.7°, internal rotation 80.0°, and external rotation 45.7° (Table 4 and Table 5).

## 4. Discussion

The optimal approach to reconstructing proximal humerus defects following sarcoma resection remains a subject of ongoing debate. Successful reconstruction must balance oncologic safety with long-term preservation of limb function. Various surgical techniques have been proposed for humeral reconstruction, each with specific advantages and limitations.

PEEK was first introduced in orthopedics in the late 1980s, initially for internal fixation and femoral stems. Its application expanded in the mid-to-late 1990s with spinal interbody fusion cages [18]. By 2005, the U.S. FDA approved a dynamic pedicle screw fixation system incorporating PEEK for lumbar fusion, signaling broader clinical acceptance. Over the past two decades, PEEK has gained widespread use in spinal and trauma devices due to its favorable mechanical and biocompatibility profiles.

Several intrinsic properties of PEEK support its use in limb-sparing oncologic reconstruction. First, its elastic modulus (~3–4 GPa) closely approximates that of cortical bone, in contrast to titanium (~110 GPa) [19]. This modulus-matching reduces stress shielding and may mitigate long-term complications such as bone resorption and implant loosening [20]. These properties are particularly relevant in young patients requiring durable reconstructions, where metallic megaprostheses often fail over time due to biomechanical mismatch [21]. Nevertheless, one implant in our series failed due to fracture at 28 months, highlighting the need for continued optimization of fatigue resistance. Future iterations may consider carbon fiber reinforcement or design modifications to improve mechanical durability while maintaining elasticity.

Second, PEEK is radiolucent and MRI-compatible, offering a clear advantage in postoperative surveillance. In oncologic cases, early detection of local recurrence is critical.

Third, the combination of 3D printing and PEEK allows for truly patient-specific reconstruction. Given the variability of bone defects after tumor resection, especially in skeletally immature or anatomically complex cases, the ability to design implants that precisely replicate the resected anatomy is invaluable. In our study, all constructs were based on contralateral mirroring and achieved sub-millimeter geometric accuracy. Prior work has demonstrated the feasibility of using 3D-printed PEEK in shoulder reconstructions with satisfactory fit and early outcomes [22]. Moreover, the lower density of PEEK (~1.3 g/cm^3^ versus ~4.5 g/cm^3^ for titanium) may reduce mechanical strain on adjacent soft tissues, potentially improving comfort and functional endurance, although this was not directly measured in our cohort.

Despite these advantages, pure PEEK is biologically inert and hydrophobic, limiting osseointegration. In osteoporotic or elderly bone, adding 20% nano-hydroxyapatite and 10% carbon fiber, or using 3D printing to create controlled porous architectures, can enhance bioactivity and tissue integration without compromising mechanical performance. In our series, additional fixation with plates and screws was employed to ensure primary stability.

When considered in context, the functional and survival outcomes in our cohort are comparable to, and in some respects exceed, those reported for conventional megaprosthetic and allograft reconstructions of the proximal humerus (Table 6). Standard modular megaprostheses (metallic endoprostheses) have historically yielded MSTS scores between 60 and 80% [23]. Osteoarticular allografts, another widely used option, offer anatomical restoration of the resected segment by preserving the graft’s joint surface. Systematic reviews report average MSTS scores of 79–82% following proximal humerus allograft-prosthetic composite (APC) reconstruction, which parallels the functional outcomes observed in our PEEK cohort [24]. However, APCs carry notable risks: union failure or graft resorption occurs in approximately 26% of cases, allograft fractures in ~10%, and overall complication rates may reach 50% [24]. These data suggest that a well-engineered polymer-based implant can serve as a viable and competitive alternative for limb-salvage reconstruction in musculoskeletal oncology.

In our series, the average forward flexion and abduction reflect a markedly limited overhead range of motion, consistent with historical outcomes of hemiarthroplasty-based megaprostheses, which typically achieve only 40–60° of flexion and 30–60° of abduction [25]. This limitation is inherent to anatomic reconstructions using either traditional tumor endoprostheses or our PEEK design, where the prosthesis often functions primarily as a spacer, particularly in the absence of a functional rotator cuff. Shoulder stability may also be compromised by axillary nerve sacrifice, leading to prosthesis subluxation or migration due to imbalanced deltoid forces [12,26,27].

To address these limitations, reverse shoulder arthroplasty (RSA) has been employed in cases of rotator cuff insufficiency, including tumor resections [12,27,28]. These semi-constrained designs medialize and distalize the center of rotation, allowing the deltoid muscle to drive shoulder function effectively [12,29,30]. In a comparative study, Grosel et al. reported superior outcomes with RSA versus hemiarthroplasty following proximal humerus tumor resection, with mean abduction reaching 85° in RSA patients compared to 28° in those receiving hemiarthroplasty [31].

The relatively modest ROM in our cohort may be attributed to the anatomic nature of the reconstruction. Our patient-specific 3D-printed PEEK prostheses restored bone length and geometry but maintained a traditional articulation with the native glenoid. Unlike reverse designs, these implants do not leverage mechanical constraints or altered biomechanics to augment deltoid-driven movement. While PEEK proved to be a safe and effective material for patient-specific reconstruction, it did not confer an intrinsic advantage in range of motion.

Our early experience with 3D-printed PEEK implants for proximal humerus reconstruction demonstrates technical feasibility and acceptable midterm outcomes. To improve implant durability, future work should focus on design optimization using finite element analysis, especially in light of the single implant fracture observed. As PEEK is biologically inert, enhancing osseointegration—via surface roughening or incorporation of bioactive fillers such as hydroxyapatite—may strengthen long-term fixation [32,33].

Patient-specific strategies are key. Pediatric patients may especially benefit from the radiolucency, weight, and customizability of PEEK. Future work should explore hybrid strategies combining the anatomical benefits of personalized PEEK implants with functional enhancements—such as reverse articulation mechanisms or integration with biological grafts—to better restore shoulder motion in this challenging patient population.

This study is limited by the small sample size, which reflects the rarity of proximal humeral sarcomas and the early clinical use of PEEK implants. Therefore, the findings should be interpreted as preliminary and not broadly generalizable. In addition, shoulder function remained limited, particularly in overhead motion, with the reconstruction serving primarily as a stable spacer rather than restoring full mobility. These functional constraints should be clearly discussed with patients preoperatively. Finally, the absence of a control group, such as metallic megaprostheses or reverse total shoulder arthroplasty, limits direct comparison, although published data suggest that RSA may provide superior range of motion through altered biomechanics rather than material-related advantages.

This study presents the first reported series of 3D-printed PEEK-based reconstructions for proximal humerus defects. The findings support PEEK as a promising alternative to metal implants and allografts, particularly due to its favorable biomechanical properties and compatibility. However, larger studies with extended follow-up are necessary to confirm long-term mechanical durability and further optimize clinical outcomes.

**Table 6:** Comparative clinical and functional outcomes of reconstruction methods.

Reconstruction Method	Implant Survival	Major Complications	Mean MSTS Score	Imaging Artifact	Customization
3D-printed PEEK (our study)	85.7%	Implant fracture (14.3%)	23.0 ± 1.6	None	Patient-specific
Metal megaprosthesis [34]	70–85%	Infection, instability, loosening	18–24	Moderate	Modular only
APC [35]	55–75%	Nonunion, graft fracture, resorption	19–26	Moderate	Limited
Osteoarticular allograft [36]	40–65%	Fracture, collapse, degeneration	18–25	None	Limited
3D-printed titanium implant [37]	80–95%	Stress shielding, stiffness Mismatch	22–26	Moderate	Patient-specific
RSA [38]	89%	Shoulder instability	23.4	Moderate	Limited

## 5. Conclusions

In conclusion, midterm outcomes of 3D-printed PEEK implants showed satisfactory implant survival and functional results, comparable to metal and allograft alternatives. PEEK’s modulus and radiolucency offer biomechanical and imaging advantages. However, limited shoulder motion and one implant fracture highlight the need for further design optimization. Enhancing osseointegration and customizing function-oriented features may improve functional results. PEEK shows strong potential as a patient-specific, limb-sparing solution in orthopedic oncology.

## Figures and Tables

**Table 1:** Patient characteristics.

No.	Age (Years)	Sex	Diagnosis	Stage	Pathologic Fracture	Follow-Up (Months)
1	41	Male	Osteosarcoma	IIB	No	112
2	26	Male	Chondrosarcoma	IIB	No	74
3	13	Male	Ewing sarcoma	IIB	No	62
4	58	Male	Chondrosarcoma	IIB	No	52
5	7	Female	Osteosarcoma	IIB	Yes	49
6	12	Male	Osteosarcoma	IIB	Yes	19
7	9	Female	Osteosarcoma	IIB	No	24

**Table 2:** Manufacturing parameters.

Parameter	Value
Printing technology	FDM
Material	Pure medical-grade PEEK
Nozzle temperature	>400 °C
Bed temperature	130 °C
Build chamber	Heated
Layer height	0.10 mm
Infill percentage	90–100%
Printing speed	30 mm/s
Post-processing	Annealing 130 °C for 2 h
Mean geometric deviation	<2 mm
Fixation method	Screw fixation to diaphyseal humerus, augmented with plate and screws
Soft tissue attachment	Integrated suture tunnels
Implant weight	~1.3 g/cm^3^

**Table 3:** Oncologic outcomes.

No.	Local Recurrence	Implant Fracture	Metastasis	Limb Salvage
1	Yes	No	None	Yes
2	Yes	No	None	Yes
3	No	Yes	None	Yes
4	No	No	None	Yes
5	No	No	None	Yes
6	No	No	None	Yes
7	No	No	None	Yes

**Table 4:** Functional outcomes.

No.	MSTS	Flexion (°)	Extension (°)	Abduction (°)	Internal Rotation (°)	External Rotation (°)
1	21	45	20	30	90	80
2	25	–	–	–	–	–
3	24	45	10	40	80	–
4	22	–	–	–	–	–
5	25	45	45	30	90	30
6	22	20	20	25	80	70
7	22	20	15	20	70	60

**Table 5:** Summary results.

Outcome	Value
Mean MSTS ± SD	23.0 ± 1.6
Mean Flexion (°)	35.0
Mean Extension (°)	22.0
Mean Abduction (°)	29.0
Mean Internal Rotation (°)	82.0
Mean External Rotation (°)	60.0
Implant Survival Rate	85.7% *(6/7 with no fracture)*
Local Recurrence Rate	28.6% *(2/7 patients)*
Limb Salvage Rate	100%

## Data Availability

The datasets analyzed during the current study are available from the corresponding authors on reasonable request.

## Abbreviations

The following abbreviations are used in this manuscript:

APC	Allograft–prosthesis composite
PEEK	Polyether ether ketone
MSTS	Musculoskeletal Tumor Society score
RSA	Reverse shoulder arthroplasty
